# The Epigenetics of Glioma Stem Cells: A Brief Overview

**DOI:** 10.3389/fonc.2020.602378

**Published:** 2020-12-02

**Authors:** Luis M. Valor, Irati Hervás-Corpión

**Affiliations:** Unidad de Investigación, Hospital Universitario Puerta del Mar, Instituto de Investigación e Innovación Biomédica de Cádiz (INiBICA), Cádiz, Spain

**Keywords:** glioblastoma, histone, DNA, methylation, acetylation, Polycomb, H3.3, HDACi

## Abstract

Glioma stem cells (GSCs) are crucial in the formation, perpetuation and recurrence of glioblastomas (GBs) due to their self-renewal and proliferation properties. Although GSCs share cellular and molecular characteristics with neural stem cells (NSCs), GSCs show unique transcriptional and epigenetic features that may explain their relevant role in GB and may constitute druggable targets for novel therapeutic approaches. In this review, we will summarize the most important findings in GSCs concerning epigenetic-dependent mechanisms.

## Introduction

GB is the most common and aggressive primary brain cancer in adults. Despite the combined clinical therapy of surgical resection, radiotherapy and chemotherapy with the first-line agent temozolomide (TMZ), the prognosis is still unfavorable, with a median overall survival of 15 months and a high risk of recurrence (>90%) (1). This ability to resist chemo- and radiotherapy can be explained by the presence of a subpopulation of cells within the perivascular and hypoxic niches of the tumor known as GSCs or brain tumor-initiating cells. The subventricular zone (SVZ) is a neurogenic niche containing NSCs and progenitor cells and is suspected to be the origin of different brain tumor types due to the generation of GSCs (2–4). GSCs share functional characteristics with NSCs, including the capacity for self-renewal and long-term proliferation required to maintain and propagate the tumor, respectively (5). In addition, GSCs exhibit other properties of cancer cells, such as angiogenesis, invasion and immunosuppression, that promote disease progression and complicate treatment (6). Cells positive for stemness markers (*e.g.*, CD133) have the ability to form tumors *in vivo* and oncospheres *in vitro* (reminiscent of neurosphere-derived NSCs) (6). In fact, understanding the hallmarks of GSCs can offer novel therapeutic strategies targeted at these cells to achieve an effective treatment for this disease.

## The Relevance of Epigenetics in the Regulation of Gene Expression in GSCs and NSCs

The nucleosome is the structural unit of chromatin and is composed of 147 bp of DNA wrapped around an octamer of histones (H2A, H2B, H3, and H4). The chromatin organization and its degree of compaction are modulated by DNA and histone covalent modifications, ATP-dependent chromatin remodeling and certain non-coding RNAs (ncRNAs). Epigenetic mechanisms contribute to the cellular hierarchy of tumoral tissue in GB (7) and are crucial to understanding tumorigenesis and response to treatment in gliomas. For example, promoter hypermethylation of the O-6-methylguanine-DNA methyltransferase (*MGMT*) gene can predict good outcomes in TMZ treatment (8, 9). Additionally, mutations in arginine 132 of the tricarboxylic acid cycle component IDH1 (or in arginine 172 of IDH2), which are associated with longer survival, induce the overproduction of the 2-hydroxybutyrate metabolite that inhibits the α-ketoglutarate-dependent activity of epigenetic enzymes such as JumonjiC histone demethylases and TET hydroxymethylases, affecting both histone and DNA methylation (10).

The gene expression profiles of GSCs resemble those of normal NSCs (11, 12), but differential gene expression patterns between both types of cells can identify a transcriptional signature that is correlated with patient survival (13); however, copy number variations only explain a small portion of such gene expression alterations and other mechanisms (*e.g.*, epigenetics) should be more relevant. For instance, changes in the patterns of DNA methylation, H3K27me3 and H3K4me3 are important in neural lineage differentiation (14–16), and a comparison of the genome-wide distribution of these and other epigenetic marks revealed important differences between GSCs and normal NSCs, affecting genes involved in neural differentiation and cancer processes (17, 18). These glioma-specific patterns of epigenetic marks can be found in DNA elements that are important for gene regulation:

- Bivalent promoters are considered a feature of embryonic stem cells (ESCs) due to their high prevalence in these cells (16, 19) and are characterized by the coexistence of epigenetic marks associated with active and repressed genes (generally H3K4me3 and H3K27me3). Genes under the control of such promoters are poised, *i.e.*, maintained in silent state but ready to be activated under appropriate external or developmental stimuli (20). Genome-wide analyses identified a high diversity of bivalent regions within GSCs, which were shown to have significantly distinct patterns compared to NSCs and ESCs (17, 21). Loss of bivalency in GSCs affected a very low number of promoters but associated with the potential activation of proto-oncogenes and genes related to transcription, and the potential repression of genes linked to cell adhesion and ion channels (17). Moreover, consistent bivalent genes across several GSCs were members of the Wnt pathway and HOX family as well as potassium channels and solute carriers that can be associated with overall survival (21).
- Enhancers often regulate cell-specific gene expression and are defined by the simultaneous occupancy of H3K27ac and H3K4me1. Although enhancer patterns are relatively conserved between GSCs and NSCs, unique GSC patterns are mainly linked to genes with functions in DNA damage response, p53 signaling and angiogenesis; prominent examples are HOX cluster genes, which acquire enhancer histone modifications in GSCs and become highly expressed despite promoter methylation (22). In contrast, NSC-specific enhancers are more associated with stem cell differentiation, apoptosis and epigenetic regulation (22).

Overall, GSCs are characterized by an impairment of differentiation due to a permanent epigenetic block that maintains the self-renewal capacity of these cells (18, 23). Nonetheless, GSCs can rapidly adapt to diverse microenvironments by modulating their transcriptomes and DNA methylomes (24), indicating that such alterations are at least partially reversible, contrary to genetic variations. Reversibility of epigenetic marks was demonstrated in reprogramming experiments of glioma cells: with the appropriate combination of transcription factors they can be reversed into an early embryonic state that was accompanied by a widespread resetting of cancer-associated DNA methylation (23). Still, this resetting was not sufficient to abolish the malignant behavior of these cancer cells, indicating that we need to decipher how epigenetic-related activities work in GSCs to explaining their malignancy. In the following sections we review the experimental evidences found in GSCs about the role of epigenetics in malignancy and potential treatments.

## The Role of Polycomb Repressive Complexes in the Maintenance of the GSC Phenotype

**Figure 1:**
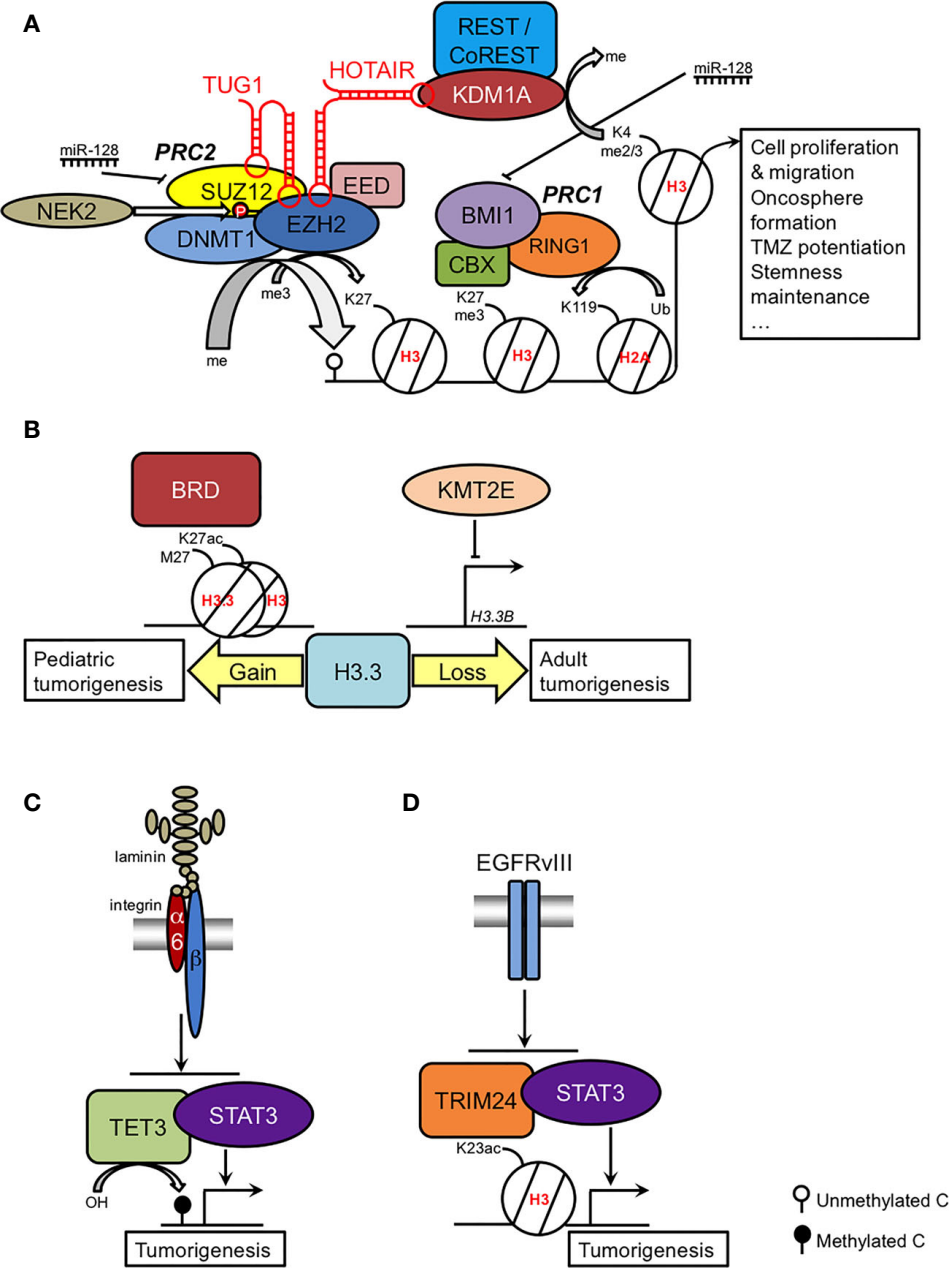
Summary of the epigenetic regulation of the GSC phenotype. **(A)** Coordinated actions and regulation of Polycomb complexes; **(B)** role of H3.3 in pediatric and adult GSCs as a result of gain and loss-of-function, respectively; **(C)** TET3-STAT regulation by the laminin-integrin signaling pathway; **(D)** H3K23ac-TRIM24-STAT regulation by the EGFRvIII signaling pathway. See main text for further details.

The Polycomb repressive complexes, essential for normal developmental processes, have been the most studied epigenetic modulators in GSCs. The most relevant findings are summarized in **Figure 1A**. Polycomb repressive complex 2 (PRC2) is necessary for neurogenesis at the SVZ (25, 26) and regulates the trimethylation of H3K27 thanks to the catalytic activity of Enhancer of Zeste Homolog 2 (EZH2), which transfers a methyl group from S-adenosyl methionine, in cooperation with Suppressor of Zeste 12 (SUZ12) and Embryonic Ectoderm Development (EED). Overexpression of EZH2 has proto-oncogenic implications in several cancers, including glioma, in which elevated EZH2 expression has been associated with high-grade disease and poor overall survival (27, 28). Moreover, EZH2 activity is required for GSC maintenance by targeting MYC expression (29). Even in cells derived from diffuse intrinsic pontine gliomas (DIPG), a brain pediatric cancer that can also affect young adults, in which the actions of EZH2 are inhibited by the H3K27M mutation, residual EZH2 activity is still retained at strong PRC2 targets to drive GSC proliferation (30). Therefore, it is not surprising that selective EZH2 inhibition can constitute a promising therapeutic approach, as treated GSCs can reduce the levels of EZH2 and H3K27me3, cell proliferation and migration, the number and diameter of oncospheres, and the growth of intracranial xenotransplanted cells in mice, reverse epithelial-mesenchymal transition (EMT), potentiate the effects of TMZ and downregulate stem cell markers while increasing the expression of differentiation markers (29, 31–33).

PRC2 activity is important for other epigenetic modifications. First, trimethylation of H3K27 is a prerequisite for histone H2A monoubiquitylation by Polycomb repressive complex 1 (PRC1) (34). Within this complex, the ring finger protein BMI1 is also a glioma stemness marker, and interference of its activity affects GSC malignancy *in vitro* and in xenotransplanted mice and enhances radiosensitivity (35–37). Second, EZH2 can recruit DNA methyltransferases (38), which explains the hypermethylation of PRC2 targets in primary GB (39, 40).

GSC characteristics display regional variations depending on the tumor niche. Whereas the regions defined by the disruption of the blood-brain barrier in angiogenesis foci were characterized by a high expression of proneural genes, an enrichment of EZH2/SUZ12/H3K27me3 targets and GSCs primarily positive to the proneural markers SOX2 and OLIG2, the hypoxic necrotic regions contained high expression of mesenchymal genes, a strong association with H2A119ub, an enrichment of BMI1 targets and GSCs primarily positive to the mesenchymal markers CD44 and YKL40 (41). Selective inhibition of either EZH2 or BMI1 was highly effective against the survival of proneural and mesenchymal GSCs, respectively. Thus, the combined strategy to abolish the activity of both PRCs can target different tumor compartments, increasing the efficacy of the therapy (41).

Research on GSCs is starting to disentangle EZH2-dependent oncogenic mechanisms. In certain GSCs, astroglial differentiation mediated by the bone morphogenic protein (BMP) and ciliary neurotrophic factor (CNTF) signaling pathways is impaired due to the silencing of the BMP receptor subtype gene *BMPR1B* by hypermethylation of its promoter, mediated by the EZH2-dependent recruitment of DNMT1 (42). Whereas incubation with BMP2 or CNTF can induce an increase in the differentiation markers GFAP or β-III tubulin in cultured NSCs and GSCs, in GSCs with impaired expression of *BMPR1B*, these trophic factors enhance proliferation (42). These pleiotropic actions are reminiscent of the role of the BMP signaling pathway in embryonic NSCs to promote either NSC proliferation or neuronal differentiation, depending on the expression of the BMP receptor subunit (43). To add more complexity to the EZH2 involvement in gliomagenesis, EZH2 can methylate non-histone proteins such as oncogenic STAT3. This association leads to enhanced activation of STAT3 to positively regulate GSC self-renewal and survival (44).

How PRC2 activity is deregulated in GSCs has been intensively explored. For instance, EZH2-dependent resistance of GSCs to radiotherapy can be explained by the transcriptional upregulation of EZH2 induced by maternal embryonic leucine zipper kinase (MELK) and activation of EZH2 through phosphorylation by NIMA-related kinase 2 (NEK2) (33, 45). Moreover, it has been proposed that dysfunction of miR-128 is an early event of gliomagenesis that increases the levels of both SUZ12 and BMI1, augmenting the histone modifications they regulate: H3K27me3 and H2AK119ub. These observations suggested a coordinated regulation of PRC1 and PRC2 activities. Therefore, restoring miR-128 expression diminishes proliferation and confers radiosensitivity (46). Additionally, EZH2 activity can be regulated by the lncRNA HOX transcript antisense RNA (HOTAIR), which is associated with poor survival in diverse cancers (47). In CD133^+^ cells, HOTAIR recruits both EZH2 and the lysine demethylase KDM1A/LSD1 to repress the tumor suppressor gene *PDCD4* (48). In addition, another lncRNA, taurine upregulated gene 1 (TUG1), also binds to EZH2 and SUZ12 to repress neuronal differentiation genes such as *BDNF*, *NGF*, and *NTF3* (49).

## The Histone Variant H3.3 in Pediatric and Adult GSCs

The histone H3 variant H3.3 can play a determinant role in pediatric GB. H3.3 is an independent replication variant that replaces the canonical histones H3.1 and H3.2 during brain development, becoming predominant in adulthood (50). H3.3 is encoded by two genes: *H3F3A* (*H3.3A*) and *H3F3B* (*H3.3B*). Mutations in *H3F3A* are present in approximately one-third of pediatric gliomas, affecting either lysine 27 (H3K27M) or glycine 34 (H3G34R/V), although the former mutation can also be found to a much lesser extent in the *HIST1H3B* (*H3C2*) gene (51–53). H3K27M is a relevant driver mutation in the pathogenesis of DIPG and is sufficient to immortalize NSCs from human embryo pons (54). In DIPG-derived cell lines, H3K27M specifically increases the acetylation of H3K27 and creates heterotopic H3K27M/H3K27ac nucleosomes that can be targeted by inhibitors of bromodomain (BRD) proteins to modulate the expression of the cell cycle arrest gene *CDKN1A*, the neuronal mature markers *TUBB3* and *MAP2*, and the Zn finger protein *GLI2* (55), a relevant downstream target of the Sonic Hedgehog pathway that is implicated in the etiology of DIPG (56) (**Figure 1B**).

In adult GB, dominant negative mutations in histone H3 are extremely rare. Instead, downregulation of the *H3F3B* gene has been reported to lead to a deficit of H3.3 function in GSCs as a result of the action of the lysine methyltransferase KMT2E (myeloid/lymphoid leukemia MLL5), maintaining the self-renewal capacity of GSCs and interfering with their differentiation (57) (**Figure 1B**). These findings suggest that H3.3 impairment in adult GB may produce similar chromatin rearrangements as the H3.3 mutation in pediatric GB, given the similar DNA methylation patterns in both types of tumors (57).

## Other Epigenetic Modulators

In addition to PRCs and H3.3, other epigenetic-related factors have been implicated in the GSC phenotype and are listed in **Table 1**.

**Table 1:** List of other epigenetic-related factors in GSC studies.

Modulator	Epigenetic action	Role and mechanisms
Helicase, lymphocyte-specific HELLS	Member of the ATP-dependent chromatin remodeling SWI2/SNF2 complexes	Maintenance of proliferation and self-renewal of GSCs through binding to the promoters of cell cycle genes, including E2F3 and MYC targets (58).
Lysine-specific demethylase KDM1A/LSD1	Demethylation of mono- and di-methylated lysines 4 and 9 of histone H3	Cell viability, oncosphere formation and tumorigenesis of intracranial xenografts. Rescue by novel inhibitors (59).
Lysine demethylase with Jumonji domain KDM6B/JMJD3	Demethylation of mono- and di-methylated lysine 27 of histone H3	Cell growth and tumorigenesis of intracranial xenografts of pediatric brainstem GSCs. Rescue by treatment with GSKJ4 (60)
Lysine methyltransferase KMT2A/MLL1	Methylation of lysine 4 of histone H3	Upregulation in GSC and in hypoxic GB. GSC growth and self-renewal (61).
Ten–Eleven Translocation TET3	Conversion of 5 mC to 5 hmC	Inhibition of self-renewal and tumorigenesis after downregulation of its repressor, the nuclear receptor NR2E1/TLX (62). In highly aggressive GSCs, maintenance of laminin-integrin *α*6 signaling pathway-dependent cell survival through TET3 interaction with STAT3 at methylated loci, leading to global increase of 5hmC levels and the upregulation of oncogenes (*e.g.*, c-Myc, surviving, BCL2-like protein BCL-XL (63) (**Figure 1C**). Inhibition of the differentiation marker GFAP (22) after TET3 translocation into the GSC nucleus.
Tripartite motif-containing protein TRIM24	Reader of histone H3 with unmethylated K4 and acetylated K23	Association with tumor grade and GB recurrence (64). In EGFRvIII-expressing glioma cells, association with increased H3K23ac and recruitment of STAT3 to promote GSC proliferation and oncosphere formation (**Figure 1D**). Rescue by treatment with EGFR inhibitor erlotinib (65).

## HDAC Inhibitors as Therapeutic Agents in GSCs

Considering that altered gene expression levels have been reported for histone deacetylases (HDACs) in GB (66, 67), most therapeutic approaches have been focused on histone deacetylase inhibitors (HDACis) due to their recognized antiproliferative effects in multiple cancer models and their benefits and tolerability in the amelioration of several neurological conditions *in vivo* at the preclinical stage; in addition, some of these compounds have been approved as therapeutic agents in other types of cancers. Histone acetylation is regulated by the opposing enzymatic activities of lysine acetyltransferases and HDACs: whereas the former enzymes transfer the acetyl group from an acetyl-CoA molecule to the lysines of the protruding histone tails (an activity that is associated with active genes), HDACs catalyze this removal, which is associated with gene repression. Inhibition of HDACs can induce cell cycle arrest, apoptosis and cellular differentiation and can interfere with cancer angiogenesis (68). One interesting target of HDACis is the phosphatase DUSP1, an inhibitor of the JNK, ERK1/2 and p38 MAPK pathways that is associated with GSC differentiation and good prognosis (69).

Among the tested HDACis in clinical trials, vorinostat/SAHA, romidepsin/FK228/FR901228 and panobinostat/LBH-589 demonstrated very limited efficacy as therapeutic agents in single therapies in both newly diagnosed and recurrent GB. However, the most promising effect of HDACis is as sensitizers to current therapeutic approaches such as radiotherapy and TMZ therapy [see (70) for a review on this topic]. Some considerations should be kept in mind to understand the potential benefits and limitations of HDACi-based treatment *in vivo*. First, acetylation increase by HDACis is not exclusive of histones (71); second, antineoplastic actions of HDACis can be achieved independently of (or in addition to) HDAC inhibition (72); third, the solubility of HDACis in water is usually poor, resulting in inefficient transport through the blood-brain barrier with oral administration (73); last, chemoresistance has been reported in long-term treatments (74). In any case, the prospects of using HDACis are still promising, and research on GSCs can help in elucidating the underlying anticancer mechanisms of HDAC inhibition and in proposing novel formulations to improve drug delivery (*e.g.*, loading these hydrophobic compounds into nanomicelles) (75). Efforts are being mainly focused on valproic acid (VPA), with proven antitumoral effects (72, 76, 77). Often administered as an anticonvulsant agent to treat epilepsy in brain tumors (78), retrospective clinical studies reported that treatment with this compound increased the overall survival of GB patients (79) although this effect was not found in other reports and still remains controversial (80, 81). VPA is capable of inducing a predifferentiation state in GSCs (74) and can be combined with other antineoplastic compounds for synergistic effects, as reported for the antimitotic paclitaxel (82). However, VPA failed to sensitize GSCs to TMZ (74), although another study reported sensitization to both TMZ and nimustine (ACNU), especially in MGMT-expressing cells (83). VPA is able to modify the DNA methylomes of GSCs (74), leading to the activation of the Wnt/*β*-catenin pathway which was related with growth inhibition, reduced migration and EMT impairment (84). This is in conflict with the suppression of the Wnt/*β*-catenin pathway by SAHA, which partially rescues the downregulation of histocompatibility complex class I and antigen-processing machinery genes, as a plausible strategy to potentiate the activation of cytotoxic T cells *in vivo* (85). Side effects have also been reported as VPA can exacerbate the unfolded protein response program, leading to protein homeostasis dysregulation and proteostasis stress in GSCs (86).

## Concluding Remarks

Research on the epigenetics of GSCs has the potential to elucidate the self-renewal and perpetuation mechanisms of these cells through the identification of the epigenetic program that governs aberrant gene activation and repression in cancer. Less known epigenetic modifications should be further explored, as they can provide further insights into tumorigenesis, as in the case of 5’-formylcytosine (5fC) and 5′-carboxylcytosine (5caC) (22). In addition, a systematic and detailed description of direct target genes of epigenetic activities is required to understand the complex mechanisms of epigenetic dysregulation in gliomas. As we have seen through this review, multiple epigenetic activities can be involved in glioma malignancy in a complex manner; therefore, the simultaneous modulation of various epigenetic activities may be highly effective, as demonstrated by the dual inhibition of HDACs and KDM1A/LSD1 (87, 88).

## Author Contributions

All authors contributed to the article and approved the submitted version.

## Funding

LV is supported by the Plan Propio INiBICA (Grant L19-07IN-CO07) and by the Programa Estatal de Generación de Conocimiento y Fortalecimiento del Sistema Español de I+D+i, financed by the Instituto de Salud Carlos III and Fondo Europeo de Desarrollo Regional 2014-2020 (Grants CP15/00180 and PI16/00722). LV is the recipient of a Miguel Servet I contract (CP15/00180) financed by the Instituto de Salud Carlos III and Fondo Social Europeo 2014-2020, Programa Estatal de Promoción del Talento y su empleabilidad en I+D+i.

## Conflict of Interest

The authors declare that the research was conducted in the absence of any commercial or financial relationships that could be construed as a potential conflict of interest.

## Abbreviations

BMP, Bone morphogenic protein; BRD, Brodomain; CNTF, Ciliary neurotrophic factor; DIPG, Diffuse intrinsic pontine gliomas; EED, Embryonic ectoderm development; EMT, Epithelial–mesenchymal transition; ESC, Embryonic stem cell; EZH2, Enhancer of Zeste homolog 2; GB, Glioblastoma; GSC, Glioma stem cell; HDAC, Histone deacetylase; HDACi, HDAC inhibitor; HOTAIR, HOX transcript antisense RNA; KAT, Lysine acetyltransferase; lncRNA, Long Non-coding RNA; MELK, Maternal embryonic leucine zipper kinase; MGMT, O-6-methylguanine-DNA methyltransferase; NEK2, NIMA-related kinase 2; NSC, Neural stem cell; PRC1/2, Polycomb repressive complex 1/2; SUZ12, Suppressor of Zeste 12; SVZ, subventricular zone; TMZ, Temozolomide; TUG1, Taurine upregulated gene 1; VPA, Valproic acid.
